# Phase III study of cisplatin with or without S-1 in patients with stage IVB, recurrent, or persistent cervical cancer

**DOI:** 10.1038/s41416-018-0206-7

**Published:** 2018-08-03

**Authors:** Yoichi Aoki, Kazunori Ochiai, Soyi Lim, Daisuke Aoki, Shoji Kamiura, Hao Lin, Noriyuki Katsumata, Soon-Do Cha, Jae-Hoon Kim, Byoung-Gie Kim, Yasuyuki Hirashima, Keiichi Fujiwara, Young-Tak Kim, Seok Mo Kim, Hyun Hoon Chung, Ting-Chang Chang, Toshiharu Kamura, Ken Takizawa, Masahiro Takeuchi, Soon-Beom Kang

**Affiliations:** 1 Department of Obstetrics and Gynecology, Graduate School of Medical Science, University of the Ryukyus, 207 Uehara Nishihara-cho, Nakagami-gun, Okinawa 903-0215 Japan; 2 Department of Obstetrics and Gynecology, The Jikei University School of Medicine, 3-19-18, Nishishimbashi, Minato-ku, Tokyo, 105-8471 Japan; 3 Department of Obstetrics and Gynecology, Gachon University Gil Medical Center, 1198 Guwol-dong, Namdong-gu, Incheon, 405-760 Korea; 4 Department of Obstetrics and Gynecology, Keio University School of Medicine, 35 Shinanomachi, Shinjyuku-ku, Tokyo, 160-8582 Japan; 5 Department of Gynecology, Osaka International Cancer Institute, 3-1-69, Otemae, Chuo-ku, Osaka, Osaka, 541-8567 Japan; 6 Department of Obstetrics and Gynecology, Kaohsiung Chang Gung Memorial Hospital and Chang Gung University College of Medicine, No. 123, Ta-Pei Road, Niao-Sung District, Kaohsiung, 83301 Taiwan; 7 Department of Breast and Medical Oncology, National Cancer Center Hospital, 5-1-1, Tsukiji, Chuo-ku, Tokyo, 104-0045 Japan; 8 Department of Obstetrics and Gynecology, Keimyung University School of Medicine, 194 Dong San Dong, Daegu, 700-712 Korea; 9 Department of Obstetrics and Gynecology, Yonsei University College of Medicine, 50-1 Yonsei-ro, Seodaemun-gu, Seoul, 03722 Korea; 10 Department of Obstetrics and Gynecology, Samsung Medical Center, Sungkyunkwan University School of Medicine, 50 Ilwon-Dong, Gangnam-gu, Seoul, 135-710 Korea; 11 Department of Gynecology, Shizuoka Cancer Center, 1007 Shimonagakubo, Nagaizumi-cho, Sunto-gun, Shizuoka, 411-8777 Japan; 12 Department of Gynecologic Oncology, Saitama Medical University International Medical Center, 1397-1, Yamane, Hidaka, Saitama, 350-1298 Japan; 13 Department of Obstetrics and Gynecology, Asan Medical Center, 388-1 Pungnap-2dong, Songpa-gu, Seoul, 138-736 Korea; 14 Department of Obstetrics and Gynecology, Chonnam National University Hwasun Hospital, 160 Ilsimri Hwasun-eup, Hwasun, Jeonnam 519-809 Korea; 15 Department of Obstetrics and Gynecology, Seoul National University College of Medicine, 50 Ilwon-Dong, Gangnam-gu, Seoul, 135-710 Korea; 16 Department of Obstetrics and Gynecology, Linkou Chang Gung Memorial Hospital and Chang Gung University Medical College, No.5, Fu-Shin Street, Kueishan County, Taoyuan, 33305 Taiwan; 17 Department of Obstetrics and Gynecology, Kurume University School of Medicine, Asahi-machi 67, Kurume, Fukuoka, 830-0011 Japan; 18 Department of Gynecology, Cancer Institute Hospital of the Japanese Foundation for Cancer Research, 3-10-6, Ariake, Koto-ku, Tokyo, 135-8550 Japan; 19 Department of Clinical Medicine (Biostatistics), Kitasato University School of Pharmacy, Shirokane 5-9-1, Minato-ku, Tokyo, 108-8641 Japan; 20 Department of Obstetrics and Gynecology, Konkuk University School of Medicine, 120-1 Neungdong-ro, Gwangjin-gu, Seoul, 05080 Korea; 21 Present Address: Department of Medical Oncology, Nippon Medical School Musashikosugi Hospital, 1-396, Kosugi-cho, Nakahara-ku, Kawasaki-shi, Kanagawa 211-8533 Japan

**Keywords:** Cervical cancer, Cervical cancer

## Abstract

**Background:**

This open-label phase III trial evaluated efficacy and safety of S-1 plus cisplatin vs. cisplatin alone as first-line chemotherapy in patients with stage IVB, recurrent, or persistent cervical cancer.

**Methods:**

Patients were randomised (1:1) to S-1 plus cisplatin (study group) or cisplatin alone (control group). In each cycle, cisplatin 50 mg/m^2^ was administered on Day 1 in both groups. S-1 was administered orally at 80–120 mg daily on Days 1–14 of a 21-day cycle in the study group. The primary endpoint was overall survival (OS).

**Results:**

A total of 375 patients were enrolled, of whom 364 (188, study group; 176, control group) received treatment. Median OS was 21.9 and 19.5 months in the study and control groups, respectively (log-rank *P* = 0.125; hazard ratio [HR] 0.84, 95% confidence interval [CI] 0.67–1.05). Median progression-free survival (PFS) was 7.3 and 4.9 months in the study and control groups, respectively (HR 0.62, 95% CI 0.48–0.80, *P* < 0.001). The adverse event (AE) rate increased in the study group despite the absence of any unexpected AEs.

**Conclusions:**

S-1 plus cisplatin did not show superiority over cisplatin alone in OS but significantly increased PFS in patients with stage IVB, recurrent, or persistent cervical cancer. Since the standard therapy has changed in the course of this study, further studies are warranted to confirm the clinical positioning of S-1 combined with cisplatin for this population.

## Introduction

Cancer of the uterine cervix is one of the most common cancers among women worldwide and is particularly prevalent in developing nations.^1^ Platinum-based combination chemotherapy has been the standard first-line chemotherapy for recurrent or advanced cervical cancer.^2^ Currently, paclitaxel and cisplatin combined with bevacizumab is considered the preferred first-line regimen in metastatic or recurrent cervical cancer.^3^ However, first-line chemotherapy options for stage IVB, recurrent, or persistent cervical cancers are limited; there are still unmet medical needs for new agents with favourable benefit-risk profiles that maintain quality of life.

In a previous study, Katsumata et al.^4^ reported promising results for S-1, an oral fluoropyrimidine-based anticancer agent, as a single agent, with an overall response rate (ORR) of 30.6% and median time to progression of 5.2 months in patients with advanced or recurrent cervical cancer. Furthermore, S-1 plus cisplatin has shown efficacy with acceptable toxicity for the treatment of gastric and lung cancer.^5,6^ Given that platinum-based combinations have been widely used for patients with advanced cervical cancer, adding S-1 to cisplatin was expected to be an effective treatment option.

We therefore compared the efficacy and safety of S-1 plus cisplatin with cisplatin alone as first-line chemotherapy in patients with stage IVB, recurrent, or persistent cervical cancer.

## Materials and methods

### Study design

This open-label, multicentre, parallel-group, randomised, phase III study, conducted at 69 institutions in Japan, Korea, and Taiwan, was designed to compare the efficacy and safety of S-1 plus cisplatin (study group) with cisplatin alone (control group) in patients with stage IVB, recurrent, or persistent cervical cancer (Supplementary Table S1). At the time this study was planned, cisplatin alone was one of the effective treatments available for this patient population.^7^ In the past, a number of cisplatin-based chemotherapies have been evaluated over cisplatin alone with respect to overall survival (OS). Although only topotecan plus cisplatin showed a survival advantage over cisplatin alone in the GOG179 trial,^8^ the use of this regimen has not become widespread because of its greater toxicity as compared to other available regimens. So far, only one study suggests a survival benefit for topotecan. The GOG204 trial, which compared different cisplatin doublets (paclitaxel, vinorelbine, gemcitabine, or topotecan), was terminated early after an interim analysis showed no improvement in OS with any regimen, compared to paclitaxel plus cisplatin.^9^ Because paclitaxel plus cisplatin was considered the most preferable treatment in terms of OS, progression-free survival (PFS), and ORR in the GOG204 trial, this combination became widely used. Paclitaxel plus carboplatin has also demonstrated noninferiority to paclitaxel plus cisplatin (JCOG0505 trial).^10^ Based on these results, paclitaxel plus platinum-based drug became frequently used; however, paclitaxel plus cisplatin failed to show superiority in OS compared with cisplatin alone in the GOG169 trial.^11^ Therefore, we considered cisplatin 50 mg/m^2^ to be the most reasonable candidate for the control arm of this study. Patients were randomly assigned (1:1) to the study group or the control group using an interactive web response system. Randomisation was stratified using a minimisation assignment based on the presence/absence of disease in a previously irradiated field, previous use of platinum-based therapy, and institution. After the investigators enrolled patients, the randomisation sequence was generated by The Kitasato Institute Clinical Trial Coordinating Center (Tokyo, Japan), independent of the study sponsor. The protocol summary is available in Supplementary Material S1. The trial was registered at ClinicalTrials.gov (NCT00770874).

### Patients

Briefly, patients had to be ≥20 years of age with histologically proven International Federation of Gynecology and Obstetrics (FIGO) stage IVB, recurrent, or persistent cervical carcinoma, with no chemotherapy or chemoradiotherapy after diagnosis of their disease. Patients were required to have adequate organ function and Eastern Cooperative Oncology Group (ECOG) performance status (PS) score of 0–1. Main exclusion criteria were known hypersensitivity to 5-fluorouracil or cisplatin, previous treatment with S-1, or disease progression during platinum-based chemotherapy or chemoradiotherapy.

### Treatment

Each treatment cycle lasted for 3 weeks, and cycles were repeated until the patient met one of the discontinuation criteria: either disease progression or unacceptable toxicity. In the study group, S-1 was administered orally twice daily from Day 1 to Day 14, followed by a 1-week rest. The initial dose of S-1 was determined according to the patient’s body surface area at the time of registration: <1.25 m^2^, 80 mg/day; ≥1.25 to <1.5 m^2^, 100 mg/day; and ≥1.5 m^2^, 120 mg/day. Cisplatin 50 mg/m^2^ was administered intravenously on Day 1 with adequate hydration. Patients in the control group received the same regimen without S-1. Detailed criteria for suspension, resumption, initiation of subsequent cycle, and dose modification of the study treatment are defined in the study protocol (Supplementary Material S2). Prophylactic use of granulocyte colony-stimulating factor was not permitted, and crossover treatment with S-1 was prohibited in the control group. If a patient developed drug-related adverse events (AEs) with either study drug, the treatment was discontinued but the patient was allowed to continue treatment with another drug.

### Study endpoints

The primary endpoint was OS, and the secondary endpoints were PFS, ORR, and safety. OS was defined as the period from the date of randomisation to death. PFS was defined as the period from the date of randomisation to disease progression or death. Survival information was obtained every 3 months after the end of treatment. Tumour imaging was performed at baseline and after every two cycles. Tumours were assessed by the investigator according to the Response Evaluation Criteria in Solid Tumors, version 1.0.^12^ AEs and laboratory values were graded according to the Common Terminology Criteria for Adverse Events, version 3.0.

### Statistical analyses

Sample size was calculated based on the survival endpoint reported in previous trials, with an expected median survival time (MST) in the control group of 9 months.^7^ An improvement in MST of at least 39% from 9 months in the control group to 12.5 months in the study group, yielding a decreased hazard ratio (HR) of 0.72 (below the point estimate for topotecan plus cisplatin, GOG179 trial), was considered clinically relevant in this population. A total of 296 events (deaths) were required for a power of 80% at a two-sided alpha 5% to detect a difference in OS using an unstratified log-rank test. Based on a planned accrual of 24 months, a minimum follow-up of 18 months and an approximate rate of 5% loss to follow-up, a total of 360 patients (180 per group) were estimated to achieve the specified number of events in the scheduled follow-up.

The efficacy endpoints of OS and PFS were assessed in the full analysis set (FAS), which comprised all patients who received the study drugs in each assigned treatment group at randomisation, even if they received a treatment different from the assigned treatment. Safety was assessed in the as-treated population (ATP), which comprised all patients who initiated treatment in both treatment groups according to the actual treatment received by the patients. ORR was evaluated in the ORR-evaluable population, which included patients in the ATP with measurable disease (at least one target lesion) at baseline.

The superiority of the study group over the control group for the primary OS analysis was tested using an unstratified log-rank test. OS in each group was summarised using Kaplan–Meier curves, and was further characterised for MST and survival probability at 12 months, with the corresponding 95% confidence intervals (CIs). In addition, HRs were estimated using the Cox proportional hazards model with only treatment as a factor. PFS was analysed in the same way as OS. ORR was compared between the two groups using Fisher’s exact test. Estimates for each group and differences were presented with associated 95% CIs.

The final statistical analysis was originally planned for 18 months after the last patient randomisation or when 296 events had occurred, whichever came later. Statistical analyses were performed with SAS software, version 9.2. All reported *P* values were two sided, and *P* values < 0.05 were considered statistically significant.

## Results

### Patient characteristics

**Fig. 1:**
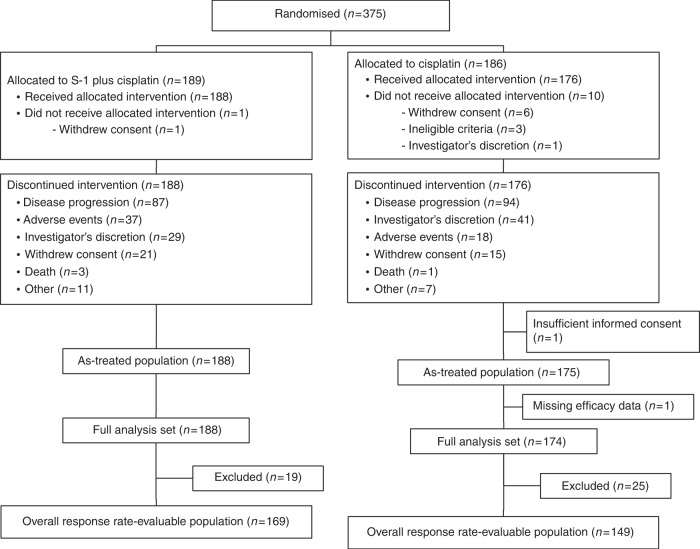
CONSORT diagram

A total of 375 patients were enrolled between September 2008 and April 2011 and randomised to the study group (189) or the control group (186); of these, 364 (188 in the study group; 176 in the control group) received study treatment (Fig. 1).

The baseline characteristics of the patients were well balanced between the two groups (Table 1). The majority of patients had recurrent disease (74.5% in the study group; 74.1% in the control group [hereinafter in this order]), a history of platinum-based therapy (64.4%; 63.8%), presence of disease in previously irradiated field (51.6%; 52.3%), and PS 0 (75.0%; 78.2%).

**Table 1:** Baseline patient characteristics

	Study group (*n* = 188)	Control group (*n* = 174)
Country of enrolment, *n* (%)		
Japan	110 (58.5)	105 (60.3)
Korea	52 (27.7)	49 (28.2)
Taiwan	26 (13.8)	20 (11.5)
Age (years)		
Median	55	52.5
Range	27**–**84	28**–**81
Body surface area (m^2^), *n* (%)		
<1.25	2 (1.1)	3 (1.7)
1.25 to <1.5	81 (43.1)	80 (46.0)
≥1.5	105 (55.9)	91 (52.3)
ECOG performance status, *n* (%)		
0	141 (75.0)	136 (78.2)
1	47 (25.0)	38 (21.8)
Disease status, *n* (%)		
IVB	22 (11.7)	27 (15.5)
Recurrent	140 (74.5)	129 (74.1)
Persistent	26 (13.8)	18 (10.3)
Histological type, *n* (%)		
Squamous	138 (73.4)	132 (75.9)
Adenosquamous	8 (4.3)	7 (4.0)
Adeno	37 (19.7)	32 (18.4)
Other	5 (2.7)	3 (1.7)
Haemoglobin (g/dL), *n* (%)		
<10.0	28 (14.9)	27 (15.5)
≥10.0	160 (85.1)	147 (84.5)
Prior radiotherapy, *n* (%)	51 (27.1)	44 (25.3)
Prior chemoradiotherapy, *n* (%)	113 (60.1)	100 (57.5)
Disease in previously irradiated field, *n* (%)		
Present	97 (51.6)	91 (52.3)
Absent	91 (48.4)	83 (47.7)
Previous history of platinum-based therapy, *n* (%)		
Yes	121 (64.4)	111 (63.8)
No	67 (35.6)	63 (36.2)

The full analysis set was used for the analysis. Study group, S-1 plus cisplatin; Control group, cisplatin

*ECOG* Eastern Cooperative Oncology Group

### Treatment

All patients discontinued the study treatment. The main reason for discontinuation was disease progression (Fig. 1).

In the ATP, the median number of cycles was 6 (range, 1–57) in the study group and 5 (range, 1–16) in the control group. The total dose of cisplatin was 255.0 mg/m^2^ in the study group and 250.0 mg/m^2^ in the control group.

### Post-treatment therapy

In the study group, 69.1% of patients received post-treatment therapy, compared with 78.7% in the control group (Supplementary Table S2). The most common first post-treatment therapy was chemotherapy (52.1% and 61.5% in the study and control groups, respectively), of which platinum-based therapy was administered to 34.0% and 44.8% of patients, respectively.

### Efficacy

**Fig. 2:**
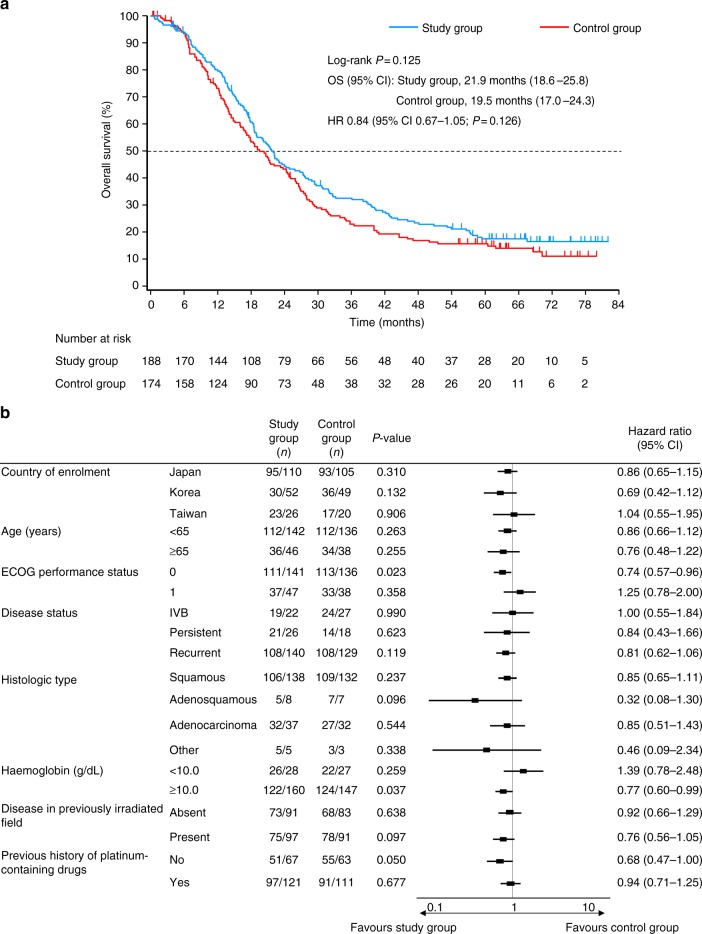
**a** Kaplan–Meier plot and **b** forest plot of hazard ratio for overall survival. Study group, S-1 plus cisplatin; Control group, cisplatin. CI confidence interval, ECOG Eastern Cooperative Oncology Group, HR hazard ratio, OS overall survival

Although the timing for the statistical analysis had been previously established, the number of events was unlikely to reach 296 in the FAS, even after 4 years of follow-up. Therefore, we conducted the final statistical analysis at the cutoff date of 30 November 2015. A total of 294 death events (148, study group; 146, control group) occurred in the FAS by the cutoff date. MST (95% CI) was 21.9 months (18.6–25.8) in the study group and 19.5 months (17.0–24.3) in the control group. There was no statistically significant difference in the OS between the two groups (log-rank *P* = 0.125, HR 0.84, 95% CI 0.67–1.05) (Fig. 2a); the 1-, 3-, and 5-year survival rates (study group vs control group) were 79.7% vs 73.6%, 32.0% vs 22.9%, and 17.5% vs 15.7%, respectively. A prespecified forest plot of HR for OS showed the favourable impact of PS 0 (HR 0.74, 95% CI 0.57–0.96, *P* = 0.023) and of haemoglobin ≥10.0 g/dL (HR 0.77, 95% CI 0.60–0.99, *P* = 0.037) on OS in the study group compared with the control group (Fig. 2b).

**Fig. 3:**
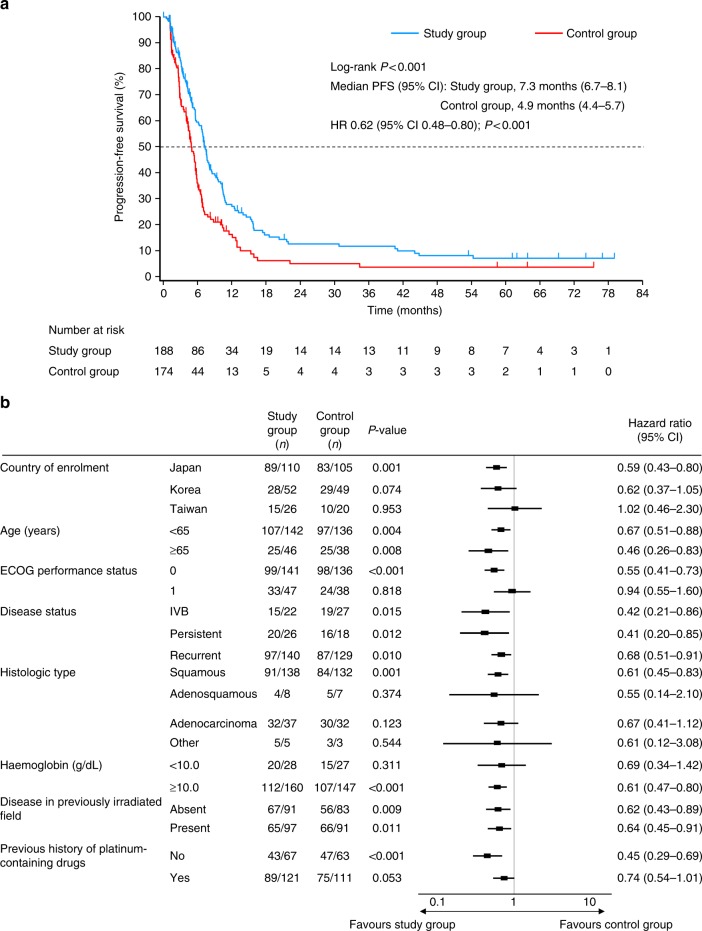
**a** Kaplan–Meier plot and **b** forest plot of hazard ratio for progression-free survival. Study group, S-1 plus cisplatin; Control group, cisplatin. CI confidence interval; ECOG Eastern Cooperative Oncology Group, HR hazard ratio, PFS progression-free survival

Median PFS was longer in the study group (7.3 months, 95% CI 6.7–8.1) compared with the control group, with a statistically significant difference (4.9 months, 95% CI 4.4–5.7) (log-rank *P* < 0.001, HR 0.62, 95% CI 0.48–0.80) (Fig. 3a). The forest plot of HR for PFS is shown in Fig. 3b.

ORR and disease control rate are shown in Table 2. ORR was significantly higher (*P* < 0.001) in the study group (43.8%, 95% CI 36.2–51.6) than in the control group (20.1%, 95% CI 14.0–27.5).

**Table 2:** Overall responses in patients receiving S-1 plus cisplatin or cisplatin alone

	Study group (*n* = 169)	Control group (*n* = 149)	*P* value^a^
Best overall response, *n* (%)			NA
Complete response	21 (12.4)	6 (4.0)	
Partial response	53 (31.4)	24 (16.1)	
Stable disease	54 (32.0)	54 (36.2)	
Progressive disease	24 (14.2)	43 (28.9)	
Unknown^b^	17 (10.1)	22 (14.8)	
Overall response rate, *n* (%)	74 (43.8)	30 (20.1)	<0.001
95% CI	36.2–51.6%	14.0–27.5%	
Disease control rate (CR + PR + SD), *n* (%)	128 (75.7)	84 (56.4)	<0.001
95% CI	68.6–82.0%	48.0–64.5%	

The overall response rate-evaluable population was used. Study group, S-1 plus cisplatin; Control group, cisplatin.

*CI* confidence interval, *CR* complete response, *NA* not applicable, *PR* partial response, *SD* stable disease.

^a^*P* value was calculated using Fisher’s exact test.

^b^Unknown contains ‘not assessable’ or ‘insufficient data’

### Safety

AEs were observed in 188/188 (100%) patients in the study group and 172/175 (98.3%) patients in the control group. The AE rate in the study group was higher than in the control group. The most frequent grade 3 or higher AEs (occurring in ≥10% of patients in either group) for the study group vs the control group were neutropaenia (52.7% vs 6.3%), anaemia (34.6% vs 17.1%), leucopaenia (32.4% vs 4.0%), anorexia (12.8% vs 2.9%), diarrhoea (11.2% vs 4.0%), hypokalaemia (11.2% vs 1.1%), and fatigue (10.6% vs 1.7%) (Table 3). The incidence of febrile neutropaenia was 2.7 and 0% of patients in the study and control groups; 1.6 and 2.8% of patients in the study and control groups had genitourinary or anal fistulas; and 8.0 and 1.1% of patients in the study and control groups had alopecia, respectively. Discontinuation of the study treatment due to AEs occurred in 13.3% and 7.4% of patients in the study and control groups, respectively. The most common toxic effect leading to study discontinuation was gastrointestinal toxicity in the study group and hypersensitivity in the control group.

**Table 3:** All-grade adverse events ≥20% patients and grade 3 and higher adverse events ≥10% patients in either group

Adverse event, *n* (%)	Study group (*n* = 188)		Control group (*n* = 174)	
	All grade	Grade ≥ 3	All grade	Grade ≥ 3
Haematological				
Neutropaenia	148 (78.7)	99 (52.7)	65 (37.1)	11 (6.3)
Anaemia	147 (78.2)	65 (34.6)	87 (49.7)	30 (17.1)
Leucopaenia	92 (48.9)	61 (32.4)	52 (29.7)	7 (4.0)
Thrombocytopaenia	90 (47.9)	17 (9.0)	26 (14.9)	5 (2.9)
Non-haematological				
Albumin decreased	46 (24.5)	6 (3.2)	23 (13.1)	2 (1.1)
Hypokalaemia	39 (20.7)	21 (11.2)	9 (5.1)	2 (1.1)
ALT (GPT) increased	38 (20.2)	3 (1.6)	23 (13.1)	2 (1.1)
Creatinine increased	38 (20.2)	4 (2.1)	39 (22.3)	2 (1.1)
Weight decreased	38 (20.2)	5 (2.7)	10 (5.7)	1 (0.6)
Clinical symptoms				
Nausea	153 (81.4)	6 (3.2)	134 (76.6)	7 (4.0)
Anorexia	141 (75.0)	24 (12.8)	105 (60.0)	5 (2.9)
Fatigue	109 (58.0)	20 (10.6)	79 (45.1)	3 (1.7)
Vomiting	103 (54.8)	12 (6.4)	71 (40.6)	4 (2.3)
Diarrhoea	94 (50.0)	21 (11.2)	51 (29.1)	7 (4.0)
Constipation	61 (32.4)	3 (1.6)	48 (27.4)	1 (0.6)
Stomatitis	59 (31.4)	7 (3.7)	16 (9.1)	0
Skin hyperpigmentation	48 (25.5)	0	2 (1.1)	0
Oedema peripheral	41 (21.8)	2 (1.1)	16 (9.1)	1 (0.6)
Peripheral sensory neuropathy	39 (20.7)	3 (1.6)	22 (12.6)	0
Pyrexia	38 (20.2)	0	20 (11.4)	1 (0.6)

The as-treated population was used for the analysis. Study group, S-1 plus cisplatin; Control group, cisplatin.

*ALT* alanine aminotransferase, *GPT* glutamic-pyruvic transaminase

Overall, 72 of 188 patients (38.3%) in the study group and 34 of 175 (19.4%) patients in the control group had serious AEs (SAEs). Fatal SAEs occurred in seven patients in the study group, of which myocardial ischaemia in one patient and disseminated intravascular coagulation and intra-abdominal haemorrhage in another patient were related to the study drugs. Three fatal SAEs were reported in the control group, but none of them were related to the study drug. No unexpected AEs were identified in either group.

## Discussion

In this study in patients with stage IVB, recurrent, or persistent cervical cancer, S-1 plus cisplatin did not improve OS over cisplatin alone. However, adding S-1 to cisplatin significantly prolonged OS in patients with better physical condition (PS 0 or haemoglobin ≥ 10.0 g/dL). Moreover, S-1 in combination with cisplatin significantly improved PFS and ORR over cisplatin alone.

There are several reasons for the lack of a significant OS benefit despite improvements in PFS and ORR. First, although the PS criteria were different (ECOG PS in our study and JCOG0505 study vs Gynecologic Oncology Group [GOG] PS in the GOG204 and GOG240), the proportion of patients with PS 0 in the cisplatin-alone group in our study was higher than that in the GOG169 and GOG179 studies with the same treatment.^8,11^ On the other hand, the proportion of patients with PS 0 in the S-1 plus cisplatin group was higher than that in the GOG204 and GOG240 studies, but similar to the JCOG0505 study.^9,10,13^ Since PS is one of the prognostic factors for cervical cancer,^14^ this might be one of the reasons for the prolonged OS observed in our study. Second, post-treatment therapy including subsequent salvage chemotherapy was used in this study. Over half of the patients in both groups received post-treatment therapy. The effect of greater use of post-treatment therapy on prolonged OS was also speculated in the JCOG0505 trial;^10^ in fact, chemotherapy was used most frequently as post-treatment therapy. As a result, median survival may have been prolonged. Third, survival post-progression (SPP) in the S-1 plus cisplatin group was 14.6 months in this study. Broglio et al.^15^ have reported that even if PFS is prolonged, OS may not show a benefit, especially for diseases with long median SPP. We thus re-calculated the statistical power of this study based on the observed OS and found the power to be 18.4%.

Notably, S-1 plus cisplatin significantly prolonged PFS to 7.3 months. Furthermore, other phase III studies have reported median PFS of 3.98 to 6.9 months with cisplatin-based doublet combinations and 8.2 months with paclitaxel and cisplatin combined with bevacizumab.^9,10,13^ Although the patients’ quality of life data are lacking in this study, S-1 plus cisplatin demonstrated a similar response in terms of PFS to these combination therapies. ORR was also significantly improved with S-1 in combination with cisplatin. Response rates of 27–48% have been obtained with cisplatin-based doublet combinations or paclitaxel and cisplatin combined with bevacizumab.^8,11,13^ Remarkably, a complete response rate of 12.4% has been obtained with S-1 plus cisplatin and was comparable to cisplatin plus paclitaxel plus bevacizumab.

Advanced cervical cancer is known to predispose patients to fistulas. Fistulas occurred in 1.6 and 6% of patients with S-1 plus cisplatin and bevacizumab combination therapy, respectively. Overall, the toxicity increased with S-1 plus cisplatin compared with cisplatin alone, but the incidence of toxicity was similar to or lower than that with cisplatin-containing doublet and triplet including bevacizumab combination therapies.^9,13^ Myelosuppression were the major AEs in patients receiving S-1 plus cisplatin; however, the incidence of grade 3 or worse adverse events of anaemia, neutropaenia, thrombocytopaenia in the S-1 plus cisplatin group, except for topotecan plus cisplatin regimen in GOG204, was similar in the GOG204 and JCOG0505 studies. The incidence of grade 3 or worse leucopoaenia and febrile neutropaenia in the cisplatin group was lower than that in the GOG204 and JCOG0505 studies.^9,10^ The S-1 plus cisplatin regimen demonstrated a tolerable safety profile in patients with stage IVB, recurrent, or persistent cervical cancer.

Patients with stage IVB, recurrent, or persistent cervical cancer are rarely curable. Therefore, selection of combination chemotherapy considering the toxicity profile and patient preference is important. For example, alopecia may worsen a patient’s quality of life and lead to interference with treatment. Alopecia occurred in 64.3% of patients treated with paclitaxel plus cisplatin.^11^ In contrast, 8% of patients experienced grade 1, but not grade 2, alopecia with S-1 plus cisplatin in this study.

We chose cisplatin 50 mg/m^2^ as the control arm of this study and considered the validity of the study results by comparing the efficacy and safety of cisplatin monotherapy in this study with those of other phase III studies. The OS of 19.5 months in the cisplatin-alone group was much longer than that reported in other studies (6.5–9.3 months) with the same therapy.^8,11^ On the other hand, PFS in the control group was similar to the PFS reported in other studies (2.8–4.5 months).^8,11,16–18^ Further, the SPP in this study was 14.6 months. According to the study by Broglio et al.^15^, if the SPP exceeds 12 months, OS may be affected, which may explain the lack of an OS advantage in the current study. As for myelosuppression, the safety results were similar to the other phase III studies.

These results suggest that S-1 plus cisplatin offers a better response than S-1 or cisplatin monotherapy and may be one of the first-line chemotherapy options in patients with FIGO stage IVB, recurrent, or persistent cervical cancer, especially for those in better physical condition (PS 0 or haemoglobin ≥ 10.0 g/dL), as seen in the forest plot analysis of OS. However, a limitation of this study is that the optimal clinical positioning of S-1 plus cisplatin among other combination chemotherapies for this population is unclear, because this study did not include combination chemotherapy as a comparator.

To further improve effectiveness, we are considering S-1 plus cisplatin with bevacizumab because overlapping toxicity is minimal. Although treatment schedule and dose are different, S-1 plus bevacizumab demonstrated acceptable toxicity in patients with metastatic colorectal cancer.^19,20^ S-1 might be a good candidate for combination therapy with bevacizumab for advanced cervical cancer.

In conclusion, S-1 plus cisplatin did not show superiority over cisplatin alone in OS. S-1 plus cisplatin may provide some benefit as first-line chemotherapy for patients with FIGO stage IVB, recurrent, or persistent cervical cancer in terms of PFS and ORR compared with cisplatin alone, although contribution to survival is unclear. Given that the standard therapy for this population has changed in the course of this study, further studies to compare with combination therapy as a comparator are warranted to confirm the clinical positioning of S-1 combined with cisplatin for this population.

## Electronic supplementary material

Supplementary file

Supplementary file

Supplementary Tables

## Data Availability

This study was conducted in compliance with Good Clinical Practice, the Declaration of Helsinki, and each country’s regulations governing the conduct of clinical trials. The study protocol was approved by the institutional review board at each site prior to the initiation of the study. All patients provided written informed consent before enrolment.
