# Supplementary material for: Phase III study of cisplatin with or without S-1 in patients with stage IVB, recurrent, or persistent cervical cancer
Source: Br J Cancer. 2018 Aug 3;119(5):530–7. doi: 10.1038/s41416-018-0206-7 (PMC6162273; doi:10.1038/s41416-018-0206-7)
Supplement: Supplementary file 1 — Supplementary file [file 41416_2018_206_MOESM1_ESM.pdf]

PROTOCOL SYNOPSIS	
**Title of Study:**<br><br>**PHASE 3 STUDY OF S-1 + CISPLATIN COMPARED WITH SINGLE-AGENT CISPLATIN IN STAGE IVB, RECURRENT OR PERSISTENT CARCINOMA OF THE CERVIX**	
**Protocol Number:**	10020380-04
**Phase:**	3
**Indication:**	Cervical cancer
**Rationale:**	• S-1 is an oral fluoropyrimidine that combines Tegafur (FT, an oral prodrug of 5-FU) with two modulators, Gimeracil (CDHP), which inhibits 5-FU degradation by DPD inhibition, and Oteracil potassium (Oxo), which inhibits 5-FU phosphorylation in the digestive tract. The drug is rationally designed to achieve an enhanced antitumor effect while decreasing adverse events.<br>• Japanese phase 2 study of S-1 in cervical cancer suggested promising response rate and good tolerability. Therefore, S-1 can be considered as a good candidate for standard therapy in cervical cancer. The recommended chemotherapy for metastatic or recurrent cervical carcinoma is either single-agent Cisplatin or Cisplatin-based combination chemotherapy. S-1 has also shown promising antitumor activity in combination with Cisplatin as well as single agent.<br>• This two-arm comparison study is designed to evaluate the efficacy and safety of S-1 in combination with Cisplatin compared with single-agent Cisplatin randomized 1:1 as first line chemotherapy in patients with stage IVB, recurrent, or persistent cervical cancer.<br>• The efficacy and safety of treatment regimen of S-1 administered orally twice daily for 21 days with a 60 mg/m^2 dose of Cisplatin administered as a 1 to 3 hour IV infusion on Day 8 is established in a Japanese phase 3 study in advanced gastric cancer. The efficacy and safety of another treatment regimen of S-1 administered for 14 days with a 60 mg/m^2 dose of Cisplatin administered on Day 1 is also investigated in a Japanese phase 1/2 study in non-small cell lung cancer.<br>Since the control arm is a 3 weeks administration schedule, the 2 weeks administration and 1 week recovery schedule is chosen for experimental arm to assure the comparability of these two arms. There is also a consideration given to the fact that the key drug for treating cervical cancer is Cisplatin, similar to lung cancer. Dose of Cisplatin in experimental arm is adjusted at 50 mg/m^2 considering that the standard dose of Cisplatin in cervical cancer is 50 mg/m^2.
**Study Objectives:**	**<u>Primary</u>**<br>• To compare the Overall Survival (OS) of S-1 + Cisplatin therapy to single-agent Cisplatin therapy in patients with stage IVB, recurrent or persistent carcinoma of the cervix<br>**<u>Secondary</u>**<br>• To compare the Progression Free Survival (PFS) of S-1+Cisplatin therapy to single-agent Cisplatin therapy<br>• To compare the Overall Response Rate (ORR) of S-1+Cisplatin therapy to single-agent Cisplatin therapy<br>• To assess the qualitative and quantitative toxicity and reversibility of toxicity of each treatment regimen

PROTOCOL SYNOPSIS	
**Design:**	This is an open-label, multicenter, multinational, two-arm, parallel randomized Phase 3 study evaluating the efficacy and safety of S-1+Cisplatin versus single-agent Cisplatin in patients with stage IVB, recurrent or persistent carcinoma of the cervix. Patients will be randomly assigned (1:1) to S-1+Cisplatin (experimental regimen, Arm A) or single-agent Cisplatin (control regimen, Arm B). Patients will be stratified by presence or absence of disease in previously irradiated field, previous history of platinum containing drugs (yes or no), and institution.<br><br>Patients will receive study medication within 8 days of randomization. Both treatment regimens will be repeated every 3 weeks until the patient meets one of the study treatment discontinuation criteria.<br><br>Crossover treatment of S-1 after discontinuation of study treatment in treatment Arm B is not permitted.<br><br>Patients will be evaluated for efficacy, including overall survival, progression-free survival, overall response rate, compliance, and for safety, where toxicity will be assessed by adverse events and laboratory evaluations. Efficacy and safety will be evaluated at selected time points.
**Duration:**	Patient accrual is planned for around 36 months. The treatment period for the study begins when the first patient is randomized and continues until when the final survival analysis take place. Assuming that the study is not stopped at the planned interim analyses or for safety reasons, the final survival analysis will take place at the end of November 2015 or the time that a total of 296 events (deaths) are observed, whichever is earlier. Patients still on treatment at the end of the study will have the option to continue treatment at the discretion of the investigator. Investigational products will be provided by sponsor until the end of study treatment for each patient.
**Inclusion Criteria:**	1. Patients with histologically proven cervical carcinoma (All histological subtype will be included).<br>2. Patients who have stage IVB, recurrent or persistent disease.<br>3. Patients who are not amenable to curative treatment with surgery and/or radiotherapy.<br>4. Patients who have not received chemotherapy or chemoradiotherapy after diagnosis of recurrent, persistent or stage IVB disease.<br>5. If the patient have received chemotherapy, radiotherapy or chemoradiotherapy as previous treatment, following interval must have elapsed from the last administration of treatment:<br>a. Chemotherapy: 21 days<br>b. Radiotherapy: 21 days[*]<br>c. Chemoradiotherapy: 42 days[*]<br>* If there have been residual disease in previously irradiated field and without disease progression since the (chemo) radiotherapy, 90 days must have elapsed after the last administration of irradiation.<br>6. Patients who have adequate hematologic, hepatic and renal functions as defined below.<br>Hemoglobin : $\geq$ 8.0 g/dL<br>Neutrophil count : $\geq$ 2,000 /mm^3<br>Platelet count : $\geq$ 100,000 /mm^3<br>Total serum bilirubin : $\leq$ 1.5 times the upper limits of normal (ULN)<br>AST (GOT), ALT (GPT) : $\leq$ 2.5 times the ULN[*]<br>Serum creatinine : $\leq$ ULN[**]<br>* If abnormal values are associated with hepatic metastasis: $\leq$ 5.0 times the ULN<br>** Or creatinine clearance: $\geq$ 50 ml/min<br>7. Patients who have an ECOG performance status : 0-1<br>8. Age: $\geq$ 20 years old<br>9. Patients who can take pills orally.<br>10. Patients who signed the written consent form.

**PROTOCOL SYNOPSIS**	
**Exclusion Criteria:**	1. Patients who have known hypersensitivity to 5-FU or Cisplatin.<br>2. Patients who are receiving concomitant treatment with drugs interacting with S-1.<br>3. Patients who are receiving concomitant treatment with drugs interacting with Cisplatin.<br>4. Patients who were administered other investigational products within 30 days before the initiation of study treatment.<br>5. Patients who were previously treated with S-1.<br>6. Patients who had received platinum-containing chemotherapy or chemoradiotherapy and whose disease progressed during the therapy.<br>7. Patients who suffer from active infection (e.g. fever ≥ 38℃).<br>8. Patients who have serious complications.<br>9. Patients with bleeding which requires hemostasis treatment.<br>10. Patients with bilateral hydronephrosis which cannot be alleviated by ureteral stents or percutaneous drainage.<br>11. Patients with uncontrolled pleural effusion and/or ascites requiring drainage at least twice a week.<br>12. Patients with symptomatic brain metastasis or history of brain metastasis.<br>13. Patients who have unmanageable bowel movement (ex. Watery stool, chronic constipation).<br>14. Patients with active double cancer.<br>15. Patients who are pregnant or lactating.<br>16. Patients who are considered to be inappropriate to the subject of this study by the investigator.
**Planned Sample Size:**	A total of 360 patients, 180/treatment arm, will be enrolled
**Investigational Product:**	S-1, Cisplatin
**Treatment Regimens:**	<u>Arm A (S-1+Cisplatin)</u><br>S-1 will be administered orally (PO), twice daily (BID) from Day 1 through Day 14 followed by a recovery period from Days 15 through Day 21. S-1 will be administered with a glass of water. Initial dose of S-1 will be determined according to the patient's body surface area (BSA). Patients with a BSA of < 1.25 m^2 will receive 80 mg/day; those with a BSA of ≥ 1.25 m^2 - < 1.5 m^2 will receive 100 mg/day; and those with a BSA of ≥ 1.5 m^2 will receive 120 mg/day. On Day 1, Cisplatin 50 mg/m^2 will be administered intravenously (IV). This regimen is to be repeated every 3 weeks.<br><u>Arm B (single-agent Cisplatin)</u><br>Cisplatin 50 mg/m^2 will be administered intravenously (IV) on Day 1. This regimen is to be repeated every 3 weeks.
**Endpoints:**	**<u>Primary</u>**<br>• Overall Survival (OS)<br>**<u>Secondary</u>**<br>• Progression-Free Survival (PFS)<br>• Overall Response Rate (ORR)<br>• Safety

PROTOCOL SYNOPSIS	
**Statistical Methods:**	Analysis Populations<br><br>**Full Analysis Set (FAS):** This population consists of all patients who are dosed, with study drug assignment designated according to initial randomization, regardless of whether patients receive a different regimen from that to which they were randomized. This will be the primary population for evaluating patient characteristics and overall survival (OS), progression free survival (PFS), and supportive analyses for these endpoints. The analysis of OS in this population will be considered as the primary analysis in the study.<br><br>**As Treated Population (ATP):** This population consists of all patients who initiated treatment in either of the two regimens with treatment assignments designated according to actual study treatment received. This will be the primary population for evaluating treatment administration, compliance and safety. The efficacy endpoints listed under the FAS section above will also be assessed in this population.<br><br>**ORR Evaluable Population:** This population consists of all ATP patients with measurable disease (at least one target lesion) at baseline. This will be the primary population for overall response rate (ORR).<br><br>Statistical Methods<br><br>The superiority test (2-sided) of experimental arm versus control arm for the primary OS analysis will be based on the unstratified log-rank test. Survival for each arm will be summarized using Kaplan Meier curves and will be further characterized in terms of the median and survival probability at 12 months, along with the corresponding 95% confidence intervals for the estimates. In addition the hazard ratio will be estimated based on Cox's proportional hazard model with only treatment as a factor.<br><br>Secondary endpoint PFS will be analyzed using the same methods as for OS.<br><br>Comparison of ORR between two treatment arms will be based on the Fisher's Exact test. Estimates of each treatment arm and its difference will be presented with associated 95% confidence intervals.<br><br>The safety evaluations will be summarized based on adverse events (AE). All patients included in the ATP will be evaluated by treatment arm in the safety analysis. All AE will be summarized (incidence) and listed by the MedDRA System Organ Class (SOC), Preferred Term (PT), CTCAE grade, and causal relationship to study medication by arm. Worst severity grade, time to event, and time to resolution will also be summarized. In addition, absolute values of laboratory parameters and their changes from baseline will be summarized by cycle. Separate summaries of SAE and Grade 3 or 4 AE will be presented.<br><br>The study drug administration profile will be summarized for each arm with respect to number of cycles taken, the dose intensity, dose modifications, dose omissions, and reason for deviations from the planned regimen.

PROTOCOL SYNOPSIS	
**Sample Size:**	Sample size considerations were based on the survival endpoint. Based on data available from multinational, randomized studies, the median survival in the control arm is expected to be 9 months. An approximately 39% improvement in median survival from 9 months in the control arm to 12.5 months in the experimental arm, yielding a reduced hazard ratio 0.72 which is below point estimate of Cisplatin + Topotecan combination therapy, will be considered clinically relevant in this population.<br><br>A total of 296 events (deaths) will be required for a two-sided unstratified log-rank test at the 5% significance and 80% power. Based on a planned accrual of 24 months, with an approximate randomization rate of 15 patients per month, a minimum follow-up of 18 months, and an approximate rate of 5% loss to follow-up, a total of 360 patients, 180 patients per arm, will be enrolled to achieve the specified number of events in the scheduled follow-up time.<br><br>Assuming that the study is not stopped, the final survival analysis will take place at the end of November 2015 or the time that a total of 296 events (deaths) are observed, whichever is earlier.
**Interim Analysis**	There will be no interim analysis to test for early demonstration of a potential superior in overall survival for the experimental arm. As such, no alpha spending has been taken into consideration for sample size calculation and the primary analysis will be performed at the 5% two-sided significance level.<br><br>A safety review will be conducted by the IDMC at 6 months after the date of randomization of the first patient. The safety review will include a comparison of the SAE profile. The IDMC will deliberate about necessity of early termination of the study due to safety profile of experimental arm, based on its results. The IDMC will make a recommendation to sponsor if needed.

TABLE 0.A STUDY SCHEDULE

Procedure	Pretreatment Period		Study Treatment Period									Post-treatment Follow-up Period		
			Cycle 1				All subsequent cycles							
	within 28 days prior to random-ization	within 14 days prior to random-ization	Day 1	Day 8[c] (Any day Days 5-11)	Day 15[c] (Any day Days 12-18)	Within 2 days prior to initiation of subsqt cycles (Any day Days -2 to 1)[d]	Day 1	Day 15[c] (Any day Days 12-18)	Within 2 days prior to initiation of subsqt cycles (Any day Days -2 to 1)[d]	Cycle 2 + All Even Cycles (Any day Day 15 ~ prior to initiation of subsqt cycle)	EOT (ASAP after trt d'c; within 2 wks of d'c due to PD)[f]	Safety F/U (30 days after last dose of study medication)	Tumor F/U[h] (every 3 months [±2 wks])	Survival F/U[i] (every 3 months [±2 wks])
Obtain Signed ICF	X													
Review Eligibility Criteria		X												
Height		X												
Performance Status		X												
Physical Examination/ Signs and Symptoms		X				X			X		X	→		
Hematology		X		X[c]	X[c]	X		X[c]	X		X	→		
Serum Chemistry		X		X[c]	X[c]	X		X[c]	X		X	→		
Urinalysis		X				X			X		X	→		
ECG	X							As	Circumstances	demand				
Vital Signs, Weight		X						As	Circumstances	demand				
BSA calculation		X				X[e]			X[e]					
Concomitant Medications			→	→	→	→	→	→	→	→	X	→		
AE Assessment[j]			→	→	→	→	→	→	→	→	X	→		
Tumor Assessment	X[a]									X	X[g]		X	
Randomization			X[b]											
Arm A and B: Cisplatin Administration			X[b]				X							
Arm A: S-1 Administration			→	→	→		→	→						
Survival Status											X	→	X	X

Abbreviations: subsqt = subsequent; ASAP = as soon as possible; d'c = discontinuation; PD = Progressive Disease; ECG = electrocardiogram; EOT = End of Treatment; F/U = follow-up; ICF = Informed Consent Form; trt = treatment with study medication.

a Baseline tumor imaging is to be performed within 28 days prior to randomization.

b Study treatment must be initiated within 8 days after randomization.

c Allowance: +/- 3 days

d Treatment recovery period may be expanded beyond Day 21 if recovery period is extended due to adverse events.

e BSA should be re-calculated if patient's weight increases or decreases by > 10% from the previous BSA calculation and is clearly not related to fluid retention.

f EOT procedures should be performed within 2 weeks from the date investigator decided to discontinue the study treatment.

g If tumor assessment was done within 6 weeks prior to the study treatment discontinuation, tumor assessment is excluded from the EOT procedures.

h Patients without PD must be followed for tumor assessment, survival, and intercurrent anti-cancer treatment during the tumor follow-up period.

i Obtain survival status (alive/dead with cause) for all treated patients until survival follow-up discontinuation or at least 18 months after randomization of the last patient.

J Adverse events should be recorded from initiation of study treatment to safety F/U. Serious adverse events should be reported from randomization to safety F/U.
