# Supplementary material for: Phase III study of cisplatin with or without S-1 in patients with stage IVB, recurrent, or persistent cervical cancer
Source: Br J Cancer. 2018 Aug 3;119(5):530–7. doi: 10.1038/s41416-018-0206-7 (PMC6162273; doi:10.1038/s41416-018-0206-7)
Supplement: Supplementary file 2 — Supplementary file [file 41416_2018_206_MOESM2_ESM.docx]

# Supplementary Material S2

## Criteria for Suspension and Resumption of S-1

S-1 administration should be withheld in case of toxicities according to the suspension criteria described below (Table 1) until recovery to the resumption criteria (Table 2).

| **Table 1 suspension criteria of S-1** | |
| --- | --- |
| Neutrophil count | < 500/mm^3^ |
| Platelet count | < 50,000 /mm^3^ |
| Infection | Infection with fever (≥ 38°C) |
| Non-hematological adverse events (AE) | Grade 3/4 Anorexia, diarrhea, stomatitis, dermatological disorder |

If AE other than listed occurs, S-1 can be suspended with the investigator’s discretion

| **Table 2 resumption criteria of S-1** | |
| --- | --- |
| Neutrophil count | ≥ 1,000 /mm^3^ |
| Platelet count | ≥ 75,000 /mm^3^ |
| Infection | No infection with fever (≥ 38°C) |
| Non-hematological AE | Anorexia, diarrhea, stomatitis, dermatological disorder; ≤ grade 2 |

If S-1 has been suspended due to AE other than listed, AE which caused suspension should be confirmed to be ≤ Grade 2 to resume.

## Criteria for Initiation of Subsequent Cycle

Before initiation of the subsequent cycle, the condition described in the following table must be confirmed (Table 3). If the patient doesn’t meet following criteria and initiation of a cycle is delayed by more than 3 weeks from the scheduled start date of the next cycle (Subsequent cycle can not be initiated by the 43rd day from the initiation of current cycle), study treatment will be discontinued.

| **Table 3 criteria for initiation of subsequent cycle** | |
| --- | --- |
| Neutrophil count | ≥ 1,500 /mm^3^ |
| Platelet count | ≥ 75,000 /mm^3^ |
| Serum creatinine | < 1.5 times the ULN |
| Infection | No infection with fever (> 38°C) |
| Non-hematological AE | Anorexia, diarrhea, stomatitis, dermatological disorder; ≤ grade 1 |

For AE other than listed, subsequent cycle can be initiated if the AE is deemed safe by the investigator.

## Dose Modification Criteria

Dose modification is not allowed in the middle of treatment cycle.

If toxicities listed below developed in the previous cycle, dose of the subsequent cycle will follow the dose modification criteria (Table 4). Dose modification for the subsequent cycle due to toxicities not listed below is also allowed if it deemed necessary.

Since this is a study in which patients will receive two chemotherapeutic agents, dose adjustment should be performed for each individual agent that is felt to be causally-related to the AE, if such a distinction can be made. If both agents are felt to be causing the AE, dose reduction should be performed for both agents. If multiple toxicities occur during a treatment cycle, the AE with the highest grade should be used as the parameter for dose adjustment.
Even in the situation which is applicable to “Not mandatory” in following table, dose reduction can be applied if investigator judge it is needed.

Dose escalation is not allowed for both S-1 and Cisplatin.

| **Table 4 Dose modification criteria** | | |
| --- | --- | --- |
| Previous cycle | S-1 | Cisplatin |
| Neutrophil count: < 500 /mm^3^  Platelet count: < 25,000 /mm^3^ | 60 mg/dose -> 50 mg/dose  50 mg/dose -> 40 mg/dose | Not mandatory |
| Grade 3/4 Diarrhea, stomatitis, dermatological disorder |  |  |
| Serum creatinine: ≥ 1.5 mg/dL |  | 50 mg/m^2^ -> 40 mg/m^2^  40 mg/m^2^ -> 30 mg/m^2^ |
| Grade 4 vomitting, anorexia related to Cisplatin | Not mandatory |  |

| **Table 5 modified dose levels** | | | |
| --- | --- | --- | --- |
| Investigational product | Initial dose | Dose reduction 1 | Dose reduction 2 |
| S-1 | 60 mg/dose | 50 mg/dose | 40 mg/dose |
|  | 50 mg/dose | 40 mg/dose | None |
|  | 40 mg/dose | None | None |
| Cisplatin | 50 mg/m^2^ | 40 mg/m^2^ | 30 mg/m^2^ |

Patients in Arm A have an option to continue the treatment with only one of the study treatment components (S-1 or Cisplatin) if AE related to one of each component requires its discontinuation. Even in this case, Criteria for initiation of subsequent cycle (Table 3) must be confirmed to continue the treatment.
