# Supplementary material for: Phase III study of cisplatin with or without S-1 in patients with stage IVB, recurrent, or persistent cervical cancer
Source: Br J Cancer. 2018 Aug 3;119(5):530–7. doi: 10.1038/s41416-018-0206-7 (PMC6162273; doi:10.1038/s41416-018-0206-7)
Supplement: Supplementary file 3 — Supplementary Tables [file 41416_2018_206_MOESM3_ESM.docx]

**Supplementary Table S1. List of Study Sites and the Latest Principal Investigators**

| Study Site | Principal Investigator |
| --- | --- |
| [Japan] |  |
| National Hospital Organization Hokkaido Cancer Center | Hidenori Kato |
| Iwate Medical University Hospital | Toru Sugiyama |
| Yamagata University Hospital | Hirohisa Kurachi |
| Niigata University Medical & Dental Hospital | Nobumichi Nishikawa |
| Jichi Medical University Hospital | Hiroyuki Fujiwara |
| Saitama Medical University International Medical Center | Keiichi Fujiwara |
| Chiba Cancer Center | Naotake Tanaka |
| Jikei University School of Medicine, Kashiwa Hospital | Hiroshi Sasaki |
| National Cancer Center Hospital | Yasuhiro Fujiwara |
| Cancer Institute Hospital | Nobuhiro Takeshima |
| Keio University Hospital | Daisuke Aoki |
| St. Marianna University School of Medicine Hospital | Kazushige Kiguchi |
| Yokohama City University Hospital | Fumiki Hirahara |
| Tokai University Hospital | Mikio Mikami |
| Kanagawa Cancer Center | Hisamori Kato |
| Shizuoka Cancer Center | Yasuyuki Hirashima |
| Nagoya University Hospital | Fumitaka Kikkawa |
| Fujita Health University Hospital | Yasuhiro Udagawa |
| Kyoto University Hospital | Tsukasa Baba |
| Osaka Medical Center for Cancer and Cardiovascular Diseases | Shoji Kamiura |
| Osaka Medical College Hospital | Yoshito Terai |
| Osaka University Hospital | Kiyoshi Yoshino |
| Wakayama Medical University Hospital | Yasushi Mabuchi |
| Hyogo Cancer Center | Satoshi Yamaguchi |
| Okayama University Hospital | Keiichiro Nakamura |
| Hiroshima University Hospital | Eiji Hirata |
| Kumamoto University Hospital | Hidetaka Katabuchi |
| Kagoshima City Hospital | Toshiaki Nakamura |
| University of the Ryukyus Hospital | Yoichi Aoki |
| National Hospital Organization Kure Medical Center and Chugoku Cancer Center | Kazuhiro Takehara |
| The Jikei University Hospital | Kyosuke Yamada |
| National Hospital Organization Shikoku Cancer center | Takayoshi Nogawa |
| Toho University Ohashi Medical Center | Kaneyuki Kubushiro |
| Chiba University Hospital | Akira Mitsuhashi |
| Kinki University Hospital | Hiroshi Hoshiai |
| Aichi Cancer Center Hospital | Shinji Kondo |
| Gunma Prefectural Cancer Center | Tatsuya Kanuma |
| Gunma University Hospital | Soichi Yamashita |
| Osaka City General Hospital | Naoki Kawamura |
| Osaka City University Hospital | Tomoyo Yasui |
| Kyushu University Hospital | Kenzo Sonoda |
| Kurume University Hospital | Kimio Ushijima |
| Oita Prefectural Hospital | Satoru Nakamura |
| Niigata Cancer Center Hospital | Shoji Kodama |
| Mie University Hospital | Tsutomu Tabata |
| Saitama Cancer Center | Harushige Yokota |
| Kitasato University Hospital | Shinpei Tsunoda |
| Osaka Rosai Hospital | Yasuhiko Shiki |
| [Taiwan] |  |
| Chang Gung Medical Foundation Linkou | Ting-Chang Chang |
| Mackay Memorial Hospital | Kung-Liahng Wang |
| National Chen Kung University Hospital | Cheng-Yang Chou |
| Chang Gung Medical Foundation Kaohsiung | Hao Lin |
| China Medical University Hospital | Lian-Shung Yeh |
| Changhua Christian Hospital | Tze-Ho Chen |
| Taipei Veterans General Hospital | Nae-Fang Twu |
| Kuo General Hospital | Yao-Tai Li |
| Chung Shan Medical University Hospital | Gin-Den Chen |
| Tri-Service General Hospital | Mu-Hsien Yu |
|  |  |
|  |  |
| [Korea] |  |
| Seoul National University Hospital | Hyun Hoon Chung |
| Ajou University Hospital | Hee-Sug Ryu |
| Gangnam Severance Hospital | Jae-Hoon Kim |
| Chonnam National University Hwasun Hospital | Seok Mo Kim |
| Keimyung University Dongsan Medical Center | Soon-Do Cha |
| Asan Medical Center | Young-Tak Kim |
| Ewha Womans University Mokdong Hospital | Seung Cheol Kim |
| Samsung Medical Center | Byoung-Gie Kim |
| Gachon University Gill Medical Center | Chan Yong Park |
| Kosin University Gospel Hospital | Won Gyu Kim |
| St. Vincent’s Hospital, The Catholic University of Korea | Dong Choon Park |

**Supplementary Table S2. Post-Treatment Therapy**

|  | Study Group  (*N*=188) | Control Group  (*N*=174) |
| --- | --- | --- |
|  | *N* (%) | *N* (%) |
| Patients with post-treatment therapy | 130 (69.1) | 137 (78.7) |
| Chemotherapy | 98 (52.1) | 107 (61.5) |
| Platinum-based therapy | 64 (34.0) | 78 (44.8) |
| Carboplatin and paclitaxel | 33 | 40 |
| Cisplatin and paclitaxel | 7 | 2 |
| Nedaplatin and irinotecan | 5 | 4 |
| Cisplatin | 5 | 7 |
| Cisplatin and topotecan | 3 | 9 |
| Nedaplatin and paclitaxel | 3 | 5 |
| Carboplatin and docetaxel | 2 | 4 |
| Carboplatin and topotecan | 2 | 0 |
| Cisplatin and 5-FU | 1 | 2 |
| Cisplatin and irinotecan | 1 | 1 |
| Carboplatin | 1 | 1 |
| Cisplatin and mitomycin | 1 | 0 |
| Cisplatin, paclitaxel and ifosfamide | 0 | 3 |
| Non-platinum-based therapy | 34 (18.1) | 29 (16.7) |
| Irinotecan | 13 | 10* |
| Paclitaxel | 7 | 4 |
| Topotecan | 5 | 3 |
| 5-FU derivatives | 3 | 8 |
| Gemcitabine | 1 | 0 |
| Others | 5 | 4 |
| Radiotherapy | 18 (9.6) | 15 (8.6) |
| Concurrent chemoradiotherapy | 7 (3.7) | 7 (4.0) |
| Surgery | 6 (3.2) | 5 (2.9) |
| Others | 1 (0.5) | 3 (1.7) |

The full analysis set was used. Study group, S-1 plus cisplatin; Control group, cisplatin.

* One patient received mitomycin with irinotecan.
